# Obesity, metabolic health, and urological disorders in adults: a nationwide population-based study

**DOI:** 10.1038/s41598-021-88165-z

**Published:** 2021-04-22

**Authors:** Jong Keun Kim, Young Goo Lee, Kyungdo Han, Jun Hyun Han

**Affiliations:** 1 Department of Urology, Hallym University Dongtan Sacred Heart Hospital, 7, Keunjaebong-gil, Hwaseong-si, Gyeonggi-do 18450 Republic of Korea; 2 Department of Urology, Hallym University Kangnam Sacred Heart Hospital, 1, Singil-ro, Yeongdeungpo-gu, Seoul, 07441 Republic of Korea; 3 Department of Statistics and Actuarial Science, Soongsil University, 369, Sangdo-Ro, Dongjak-gu, Seoul, 06978 Republic of Korea

**Keywords:** Health care, Medical research, Risk factors, Signs and symptoms, Urology

## Abstract

We evaluate the risks of various urological disorders that require treatments according to obesity and metabolic health status using a nationwide dataset of the Korean population. 3,969,788 patients who had undergone health examinations were enrolled. Participants were classified as “obese” (O) or “non-obese” (NO) using a BMI cut-off of 25 kg/m2. People who developed ≥ 1 metabolic disease component in the index year were considered “metabolically unhealthy” (MU), while those with none were considered “metabolically healthy” (MH). There were classified into the MHNO, MUNO, MHO, and MUO group. In BPH, chronic renal disease, neurogenic bladder, any medication related to voiding dysfunction, alpha-blocker, and antidiuretics, age and gender-adjusted hazard ratio (HR) was highest in MUO, but higher in MUNO than in MHO. In stress incontinence, prostate surgery, and 5alpha-reductase, HR increased in the order of MUNO, MHO, and MUO. In prostatitis, anti-incontinence surgery, and cystocele repair, HR was higher in MHO than MUNO and MUO. In cystitis, cystostomy, and anticholinergics, HR was higher in MUNO and MUO than MHO. In conclusion, obesity and metabolic health were individually or collaboratively involved in urological disorders related to voiding dysfunction. Metabolic healthy obesity needs to be distinguished in the diagnosis and treatment of urological disorders.

## Introduction

The reported global prevalence of metabolic syndrome is < 10% to 84%, according to how the syndrome is defined and the population that is studied (race/ethnicity, sex, age, location, and environmental factors)^1,2^. Impaired glucose tolerance, dyslipidemia, hypertension, and abdominal obesity are shared pathophysiological contributors to metabolic syndrome and lower urinary tract symptoms (LUTS), which are both multifactorial conditions with shared risk factors^1–4^. The components of metabolic syndrome in men over 60 years are closely associated with LUTS, which frequently develop as a result of increased tone of smooth muscle in the prostate and bladder and enlarged prostate volume^5^. In men, weight gain, a large waist circumference, and a high body mass index (BMI) increase the risk of LUTS and exacerbate their progression^6^. Women with high BMI and diabetes are also at an increased risk for urinary incontinence and overactive bladder^7,8^. Previous studies showed that people in the metabolic healthy obese (MHO) or metabolic unhealthy non-obese (MUNO) group have different risks in terms of the incidence of type 2 diabetes, cardiovascular diseases, and mortality^9–11^. Thus, clinicians should assess the risk of MHO and MUNO individuals for various urological disorders related to voiding dysfunction that require proper treatment. However, no large-scale study has yet investigated this issue. Therefore, using a nationwide dataset of the Korean population, we classified individuals into 4 groups—metabolic healthy non-obese (MHNO), MUNO, MHO, and metabolic unhealthy obese (MUO)—and aimed to evaluate the associations of obesity and metabolic health status with the risk of various urological disorders related to voiding dysfunction requiring treatment.

## Results

### Baseline characteristics according to BMI and metabolic health status

**Figure 1:**
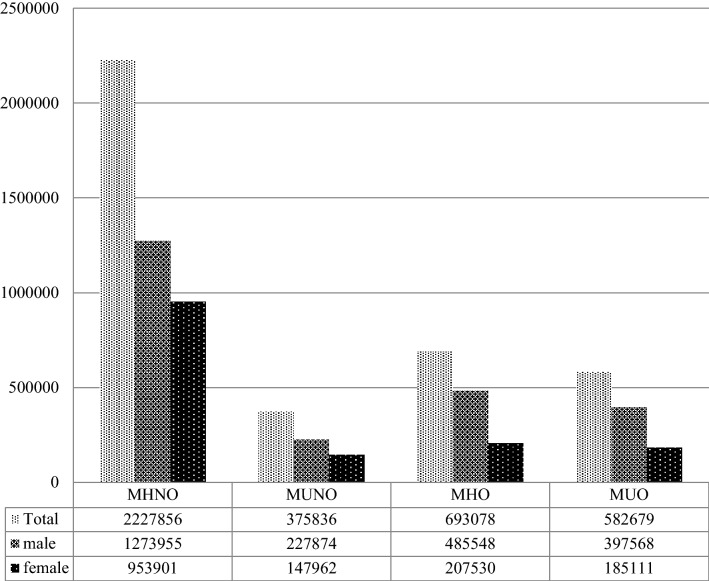
Distribution of study population according to BMI and metabolic health status. Of the 3,879,449 participants, 2,227,856 (57.4%), 375,836 (9.7%), 693,078(17.9%) and 582,679 (15.0%) subjects were classified as having the MHNO, MUNO, MHO, and MUO phenotypes, respectively.

Of the 3,879,449 participants, 2,227,856 (57.4%), 375,836 (9.7%), 693,078 (17.9%) and 582,679 (15.0%) subjects were defined as having the MHNO, MUNO, MHO, and MUO phenotypes, respectively (Table 1) (Fig. 1). The mean BMI was 21.8 ± 2 kg/m^2^, 22.9 ± 1.6 kg/m^2^, 26.8 ± 1.9 kg/m^2^, and 27.9 ± 2.4 kg/m^2^ in participants with the MHNO, MUNO, MHO, and MUO phenotypes, respectively (Table 1). As expected, the values for metabolic disease components (total cholesterol levels, fasting glucose, and systolic and diastolic BP) were higher in the MUNO and MUO groups than in the metabolically healthy groups (*P* < 0.001) (Table 1). Significant differences were also found among the groups in age, sex, family history of relevant conditions, smoking/alcohol consumption, income, and exercise (*P* < 0.001) (Table 1).

**Table 1:** Characteristics of subjects according to body mass index and metabolic health status.

	MHNO	MUNO	MHO	MUO
N (%)	2,227,856 (57.4%)	375,836 (9.7%)	693,078 (17.9%)	582,679 (15.0%)
**Age**				
Mean ± SD	43.4 ± 13.8	56.5 ± 12.8	44 ± 12.5	51.3 ± 13.2
20–39	937,385 (42.08)	38,073 (10.13)	271,340 (39.15)	122,878 (21.09)
40–64	1,096,397 (49.21)	229,140 (60.97)	373,469 (53.89)	355,449 (61)
65-	194,074 (8.71)	108,623 (28.9)	48,269 (6.96)	104,352 (17.91)
Sex, male	1,273,955 (57.18)	227,874 (60.63)	485,548 (70.06)	397,568 (68.23)
Height	164.9 ± 8.9	162.7 ± 9.5	166.1 ± 9.3	165.1 ± 9.8
Weight	59.5 ± 8.7	60.9 ± 8.6	74.2 ± 9.6	76.2 ± 11.3
Body mass index	21.8 ± 2	22.9 ± 1.6	26.8 ± 1.9	27.9 ± 2.4
Systolic blood pressure	118.7 ± 13.8	130.9 ± 15.1	123.4 ± 13.4	132.3 ± 14.6
Diastolic blood pressure	74.2 ± 9.4	80.5 ± 10.1	77.4 ± 9.3	82.3 ± 10.1
Glucose	92.3 ± 17.6	113.2 ± 36.5	93.3 ± 16.2	110.3 ± 32.1
Total cholesterol	189 ± 37.4	202.5 ± 51.4	200.1 ± 37.4	206.6 ± 47.3
**Family history**				
Hypertension	199,757 (13.54)	44,390 (18.49)	71,461 (15.2)	79,244 (20.34)
Diabetes mellitus	165,645 (11.23)	34,963 (14.57)	61,074 (13)	59,285 (15.23)
Heart disease	65,594 (4.45)	11,964 (4.99)	22,121 (4.71)	20,522 (5.28)
Stroke	108,378 (7.35)	25,407 (10.59)	37,714 (8.03)	38,775 (9.96)
**Smoke**				
Non	1,287,574 (57.79)	205,526 (54.69)	346,521 (50)	288,227 (49.47)
Ex	291,489 (13.08)	64,133 (17.06)	122,202 (17.63)	112,755 (19.35)
Current	648,793 (29.12)	106,177 (28.25)	224,355 (32.37)	181,697 (31.18)
**Drink**				
Non	1,059,652 (47.56)	204,121 (54.31)	295,215 (42.59)	273,198 (46.89)
1–2 times/week	876,133 (39.33)	104,810 (27.89)	295,771 (42.67)	204,870 (35.16)
≥ 3 times/week	292,071 (13.11)	66,905 (17.8)	102,092 (14.73)	104,611 (17.95)
**Exercise**				
Non	1,081,004 (48.52)	196,342 (52.24)	298,217 (43.03)	281,384 (48.29)
1–4 times/week	958,727 (43.03)	138,788 (36.93)	328,435 (47.39)	245,101 (42.06)
≥ 5 times/week	188,125 (8.44)	40,706 (10.83)	66,426 (9.58)	56,194 (9.64)
Income, lower 20%	390,329 (17.52)	65,640 (17.47)	118,014 (17.03)	95,804 (16.44)
**Hypertension**	340,824 (15.3)	237,933 (63.31)	151,406 (21.85)	355,796 (61.06)
Code/medication	213,568 (9.59)	190,077 (50.57)	88,996 (12.84)	262,509 (45.05)
Lab	186,127 (8.35)	106,218 (28.26)	92,021 (13.28)	188,540 (32.36)
**Diabetes**	71,148 (3.19)	98,832 (26.3)	22,823 (3.29)	132,325 (22.71)
Code/medication	37,074 (1.66)	71,492 (19.02)	10,531 (1.52)	86,823 (14.9)
Lab	54,375 (2.44)	67,431 (17.94)	18,032 (2.6)	93,824 (16.1)
**Dyslipidemia**	174,264 (7.82)	174,328 (46.38)	87,648 (12.65)	226,505 (38.87)
Code/medication	23,925 (1.07)	141,558 (37.66)	6,312 (0.91)	153,472 (26.34)
Lab	162,182 (7.28)	69,364 (18.46)	84,245 (12.16)	111,280 (19.1)

Data are expressed as the means ± SD, (%).

*MHNO* metabolically healthy non-obese; *MHO* metabolically healthy obese; *MUNO* metabolically unhealthy non-obese; *MUO* metabolically unhealthy obese.

### Diagnosis related to urological disorders according to BMI and metabolic health status

After adjusting for age and sex, the hazard ratio (HR) (95% CI) of a diagnosis of BPH was 1.185 (1.176, 1.195) for the MUNO group, 1.132 (1.123, 1.141) for the MHO group, and 1.293 (1.284, 1.303) for the MUO group, compared to those in the MHNO group. For prostatitis, the age- and sex- adjusted HRs (95% CI) were 1.013 (0.999, 1.026) for the MUNO group, 1.025 (1.015, 1.035) for the MHO group, and 1.016 (1.006, 1.027) for the MUO group, compared to the MHNO group. For cystitis, the age- and sex-adjusted HRs (95% CI) were 1.085 (1.077, 1.094) for the MUNO group, 0.953 (0.947, 0.96) for the MHO group, and 1.102 (1.094, 1.109) for the MUO group, compared to the MHNO group. For stress incontinence, the age- and sex-adjusted HRs (95% CI) were 1.015 (0.995, 1.037) for the MUNO group, 1.126 (1.105, 1.148) for the MHO group, and 1.153 (1.132, 1.174) for the MUO group, compared to those in the MHNO group. For chronic renal disease, the age- and sex- adjusted HRs (95% CI) were 2.055 (2.017, 2.094) for the MUNO group, 1.114 (1.089, 1.139) for the MHO group, and 2.084 (2.048, 2.12) for the MUO group, compared to those in the MHNO group. For neurogenic bladder, the age-and sex- adjusted HRs (95% CI) were 1.098 (1.088, 1.108) for the MUNO group, 1.025 (1.017, 1.033) for the MHO group, and 1.133 (1.124, 1.142) for the MUO group, compared to the MHNO group (Table 2).

**Table 2:** Diagnosis related to urological disorders according to BMI and metabolic health status.

	N	Event	Crude HR	Age, Sex adjusted HR
**BPH**				
MHNO	1,273,955	235,576	1 (ref.)	1 (ref.)
MUNO	227,874	77,025	2.092 (2.075, 2.109)	1.185 (1.176, 1.195)
MHO	485,548	86,476	0.951 (0.944, 0.958)	1.132 (1.123, 1.141)
MUO	397,568	109,122	1.58 (1.568, 1.591)	1.293 (1.284, 1.303)
**Prostatitis**				
MHNO	1,273,955	143,372	1 (ref.)	1 (ref.)
MUNO	227,874	28,340	1.139 (1.125, 1.154)	1.013 (0.999, 1.026)
MHO	485,548	55,285	1.005 (0.995, 1.015)	1.025 (1.015, 1.035)
MUO	397,568	47,618	1.07 (1.059, 1.081)	1.016 (1.006, 1.027)
**Cystitis**				
MHNO	2,227,856	428,306	1 (ref.)	1 (ref.)
MUNO	375,836	80,526	1.153 (1.144, 1.161)	1.085 (1.077, 1.094)
MHO	693,078	107,025	0.78 (0.775, 0.785)	0.953 (0.947, 0.96)
MUO	582,679	110,585	0.986 (0.98, 0.993)	1.102 (1.094, 1.109)
**Stress incontinence**				
MHNO	2,227,856	45,146	1 (ref.)	1 (ref.)
MUNO	375,836	12,732	1.714 (1.681, 1.748)	1.015 (0.995, 1.037)
MHO	693,078	14,267	1.011 (0.992, 1.03)	1.126 (1.105, 1.148)
MUO	582,679	17,467	1.487 (1.462, 1.514)	1.153 (1.132, 1.174)
**Chronic renal disease**				
MHNO	2,227,856	29,960	1 (ref.)	1 (ref.)
MUNO	375,836	18,967	3.896 (3.826, 3.968)	2.055 (2.017, 2.094)
MHO	693,078	10,472	1.119 (1.094, 1.144)	1.114 (1.089, 1.139)
MUO	582,679	23,848	3.09 (3.038, 3.143)	2.084 (2.048, 2.12)
**Neurogenic bladder**				
MHNO	2,227,856	248,921	1 (ref.)	1 (ref.)
MUNO	375,836	68,025	1.718 (1.704, 1.733)	1.098 (1.088, 1.108)
MHO	693,078	81,197	1.046 (1.038, 1.055)	1.025 (1.017, 1.033)
MUO	582,679	94,670	1.499 (1.488, 1.51)	1.133 (1.124, 1.142)

Data are expressed as HRs (95% confidence interval).

*BPH* benign prostate hyperplasia; *MHNO* metabolically healthy non-obese; *MHO* metabolically healthy obese; *MUNO* metabolically unhealthy nonobese; *MUO* metabolically unhealthy obese.

In BPH, chronic renal disease, neurogenic bladder, age and gender-adjusted HR was highest in MUO, but higher in MUNO than in MHO. In stress incontinence, HR increased in the order of MUNO, MHO, and MUO. In prostatitis, HR was higher in MHO than MUNO and MUO. In cystitis, HR was higher in MUNO and MUO than MHO. Rather, the HR of MHO was 0.953, which is lower than that of the control MHNO.

### Surgery related to urological disorders according to BMI and metabolic health status

After adjusting the age and sex, the HR (95% CI) for prostate surgery was 1.014 (0.958, 1.073) for the MUNO group, 1.118 (1.054, 1.187) for the MHO group, and 1.22 (1.159, 1.284) for the MUO group, compared to the MHNO group. For prostate hyperthermia, the age- and sex-adjusted HRs (95% CI) were 1.002 (0.847, 1.187) for the MUNO group, 1.089 (0.949, 1.249) for the MHO group, and 1.046 (0.909, 1.204) for the MUO group, compared to the MHNO group. For anti-incontinence surgery, the age- and sex-adjusted HRs (95% CI) were 0.916 (0.869, 0.966) in the MUNO group, 1.493 (1.438, 1.549) in the MHO group, and 1.111 (1.063, 1.162) in the MUO group, compared to the MHNO group. For cystocele repair, the age- and sex-adjusted HRs (95% CI) were 1.224 (1.023, 1.464) in the MUNO group, 1.317 (1.114, 1.557) in the MHO group, and 1.232 (1.043, 1.456) in the MUO group, compared to those in the MHNO group. For cystostomy, the age- and sex-adjusted HRs (95% CI) were 1.503 (1.138, 1.984) in the MUNO group, 0.988 (0.721, 1.353) in the MHO group, and 1.894 (1.494, 2.401) in the MUO group, compared to the MHNO group (Table 3).

**Table 3:** Surgery related to urological disorders according to BMI and metabolic health status.

	N	Event	Crude HR	Age, Sex adjusted HR
**Prostate surgery**				
MHNO	1,273,955	4638	1 (ref.)	1 (ref.)
MUNO	227,874	1655	2.046 (1.934, 2.164)	1.014 (0.958, 1.073)
MHO	485,548	1447	0.813 (0.766, 0.862)	1.118 (1.054, 1.187)
MUO	397,568	2185	1.513 (1.438, 1.592)	1.22 (1.159, 1.284)
**Prostate hyperthermia**				
MHNO	1,273,955	727	1 (ref.)	1 (ref.)
MUNO	227,874	173	1.359 (1.151, 1.604)	1.002 (0.847, 1.187)
MHO	485,548	284	1.019 (0.888, 1.169)	1.089 (0.949, 1.249)
MUO	397,568	269	1.188 (1.033, 1.366)	1.046 (0.909, 1.204)
**Anti-incontinence surgery**				
MHNO	953,901	11,341	1 (ref.)	1 (ref.)
MUNO	147,962	1802	1.04 (0.99, 1.093)	0.916 (0.869, 0.966)
MHO	207,530	3835	1.557 (1.501, 1.615)	1.493 (1.438, 1.549)
MUO	185,111	2719	1.241 (1.191, 1.295)	1.111 (1.063, 1.162)
**Cystocele repair**				
MHNO	953,901	538	1 (ref.)	1 (ref.)
MUNO	147,962	183	2.219 (1.876, 2.625)	1.224 (1.023, 1.464)
MHO	207,530	187	1.592 (1.348, 1.88)	1.317 (1.114, 1.557)
MUO	185,111	216	2.064 (1.763, 2.418)	1.232 (1.043, 1.456)
**Cystostomy**				
MHNO	2,227,856	163	1 (ref.)	1 (ref.)
MUNO	375,836	76	2.812 (2.142, 3.692)	1.503 (1.138, 1.984)
MHO	693,078	51	1.002 (0.731, 1.372)	0.988 (0.721, 1.353)
MUO	582,679	119	2.794 (2.206, 3.54)	1.894 (1.494, 2.401)

Data are expressed as HRs (95% confidence interval).

*MHNO* metabolically healthy non-obese; *MHO* metabolically healthy obese; *MUNO* metabolically unhealthy nonobese; *MUO* metabolically unhealthy obese.

In prostate surgery, HR increased in the order of MUNO, MHO, and MUO. In anti-incontinence surgery, and cystocele repair, HR was higher in MHO than MUNO and MUO. In anti-incontinence surgery, the HR of MUNO is 0.916, which is lower than the control MHNO. In cystostomy, HR was higher in MUNO and MUO than MHNO.

### Medication related to voiding dysfunction according to BMI and metabolic health status

For any medication related to voiding dysfunction, the age- and sex-adjusted HRs (95% CI) were 1.087 (1.079, 1.094) in the MUNO group, 1.029 (1.022, 1.035) in the MHO group, and 1.139 (1.132, 1.145) in the MUO group, compared to the MHNO group. For alpha-blockers, the age- and sex-adjusted HRs (95% CI) were 1.147 (1.138, 1.156) for the MUNO group, 1.092 (1.084, 1.1) for the MHO group, and 1.219 (1.21, 1.227) for the MUO group, compared to the MHNO group. For anticholinergics, the age- and sex-adjusted HRs (95% CI) were 1.052 (1.042, 1.062) for the MUNO group, 0.973 (0.964, 0.982) for the MHO group, and 1.093 (1.083, 1.102) for the MUO group, compared to the MHNO group. For 5-alpha-reductase, the age- and sex-adjusted HRs (95% CI) were 1.16 (1.145, 1.174) for the MUNO group, 1.162 (1.148, 1.177) for the MHO group, and 1.304 (1.289, 1.318) for the MUO group, compared to the MHNO group. For antidiuretics, the age- and sex-adjusted HRs (95% CI) were 1.184 (1.153, 1.216) for the MUNO group, 1.108 (1.077, 1.14) for the MHO group, and 1.295 (1.264, 1.327) for the MUO group, compared to the MHNO group (Table 4).

**Table 4:** Medication related to voiding dysfunction according to BMI and metabolic health status.

	N	Event	Crude HR	Age, Sex adjusted HR
**Any medication related to voiding dysfunction**				
MHNO	2,227,856	400,074	1 (ref.)	1 (ref.)
MUNO	375,836	112,873	1.842 (1.83, 1.854)	1.087 (1.079, 1.094)
MHO	693,078	133,251	1.073 (1.066, 1.08)	1.029 (1.022, 1.035)
MUO	582,679	157,409	1.596 (1.587, 1.605)	1.139 (1.132, 1.145)
**Alpha-blockers**				
MHNO	2,227,856	262,421	1 (ref.)	1 (ref.)
MUNO	375,836	82,716	2.026 (2.01, 2.042)	1.147 (1.138, 1.156)
MHO	693,078	94,165	1.159 (1.151, 1.168)	1.092 (1.084, 1.1)
MUO	582,679	115,704	1.773 (1.761, 1.785)	1.219 (1.21, 1.227)
**Anticholinergics**				
MHNO	2,227,856	208,269	1 (ref.)	1 (ref.)
MUNO	375,836	56,289	1.68 (1.664, 1.695)	1.052 (1.042, 1.062)
MHO	693,078	61,195	0.938 (0.929, 0.946)	0.973 (0.964, 0.982)
MUO	582,679	75,224	1.409 (1.397, 1.42)	1.093 (1.083, 1.102)
**5 alpha-reductase (male)**				
MHNO	1,273,955	92,503	1 (ref.)	1 (ref.)
MUNO	227,874	34,091	2.204 (2.177, 2.231)	1.16 (1.145, 1.174)
MHO	485,548	32,397	0.91 (0.899, 0.922)	1.162 (1.148, 1.177)
MUO	397,568	45,279	1.609 (1.591, 1.627)	1.304 (1.289, 1.318)
**Antidiuretics**				
MHNO	2,227,856	19,239	1 (ref.)	1 (ref.)
MUNO	375,836	7741	2.449 (2.385, 2.514)	1.184 (1.153, 1.216)
MHO	693,078	6482	1.078 (1.048, 1.108)	1.108 (1.077, 1.14)
MUO	582,679	9972	1.993 (1.945, 2.042)	1.295 (1.264, 1.327)

Data are expressed as HRs (95% confidence interval).

*MHNO* metabolically healthy non-obese; *MHO* metabolically healthy obese; *MUNO* metabolically unhealthy nonobese; *MUO* metabolically unhealthy obese.

In any medication related to voiding dysfunction, alpha-blocker, and antidiuretics, HR was highest in MUO, but higher in MUNO than in MHO. In 5alpha-reductase, HR increased in the order of MUNO, MHO, and MUO. In anticholinergics, HR was higher in MUNO and MUO than MHNO, the HR of MHO is 0.973, which is lower than the control MHNO.

## Discussion

Metabolic syndrome is a complex pathologic condition leading to an increased risk of diabetes and death from cardiovascular and non-cardiovascular causes^12^. Obesity is a common comorbidity associated with LUTS (e.g., frequency, incomplete emptying, intermittency, hesitancy, straining, weak stream, nocturia, and urgency), which are highly prevalent in older men. Abdominal obesity, which tends to co-occur with limited physical activity, may have an association with higher levels of sympathetic activity and is related to the severity of LUTS and the development and progression of BPH to benign prostatic obstruction^2,13^. Diminished physical activity also has a causal relationship with hyperinsulinemia and abnormal blood glucose levels. Of particular concern, extended durations of high serum glucose levels selectively exert neurotoxic effects on parasympathetic neurons, with implications for the tone of smooth muscle of the bladder^2,13^. In a cross-sectional study of National Health and Nutrition Examination Survey data, older adults (> 60 years of age) with a BMI exceeding 25 kg/m^2^ showed more severe LUTS^14^. Evidence suggests an association between metabolic syndrome and LUTS due to BPH^15,16^. It has been found that insulin resistance and secondary hyperinsulinemia augment the risk of BPH^17^, and case series have confirmed that metabolic syndrome is associated with the growth rate of prostate volume^18,19^. Bunn et al., in a systematic review, concluded that women with metabolic syndrome may be at an elevated risk for overactive bladder^20^.

In the present study, the authors analyzed the pattern of urological disorders according to obesity and metabolic health status in three areas: diagnostic disease codes, surgical procedures, and medications related to voiding dysfunction. For BPH, chronic renal disease, and neurogenic bladder, the age and sex-adjusted HR was highest in the MUO group, but higher in the MUNO group than in the MHO group. The same pattern was seen for medications related to voiding dysfunction, alpha-blockers, and antidiuretics. This suggests that metabolic health may have a greater effect on these disease and medication than obesity itself. Multiple studies have demonstrated a link between the component of metabolic syndrome and BPH/LUTS complex^2–4^. Factors including autonomic hyperactivity, hyperinsulinemia, inflammation, and obesity may play a role in the causes of metabolic symdrome and BPH/LUTS^1,21^. Chen et al.^22^ showed that patients with metabolic syndrome have a 2.5-fold higher risk of developing chronic renal disease. Studies suggest that the renal fibrosis seen in metabolic syndrome might be caused by a constellation of insulin resistance, hypertension, dyslipidemias and inflammation, and result in a heightened expression of adipocytokines, angiotensin and inflammatory cytokines such as interleukin-6 and tumor necrosis factor-alpha^22^. In this study, all types of neurogenic bladder were included in the analysis, but if we only look at the neurogenic bladder by representative SCI (spinal cord injury) patient, the inactivity imposed by SCI leads to an increase in BMI for many people and consequently an increased incidence of central adiposity and insulin resistance, components of the metabolic syndrome. This in turn leads to an increased risk of diabetes, an independent risk factor for mortality in people with SCI^23^. Obesity is also known to be associated with an increased risk of urinary tract infection (UTI) in men and women^24,25^. The obese were up to 2.5 times more likely to be diagnosed with a UTI than were the nonobese^24^.

For stress incontinence, prostatic surgery, and 5-alpha-reductase, the lowest age and sex-adjusted HR was found for the MUNO group, followed in increasing order by the MHO and MUO groups, indicating the cumulative effects of obesity and metabolic health. For prostatitis, anti-incontinence surgery, and cystocele repair, the age- and sex-adjusted HR was higher in the MHO group than in the MUNO and MUO groups. Increased intra-abdominal pressure owing to obesity appears to be the common pathophysiologic factor in stress urinary incontinence and pelvic organ prolapse for women and midurethral sling surgery is large effective in the obese population^26,27^. The authors believe that obesity itself is more influential regarding the choice of surgical treatment methods for stress urinary incontinence and cystocele than metabolic health, and that medical control is preferred in the MUO group over surgical treatment. Zhang et al.^28^ reported that major lifestyle factors such as obesity, smoking and hypertension were not associated with chronic prostatitis/chronic pelvic pain syndrome risk in the large cohort study. The etiology of chronic prostatitis/chronic pelvic pain syndrome remains unknown. For cystitis, cystostomy, and anticholinergics, the age and sex-adjusted HR was higher in the MUNO and MUO groups than in the MHO group, implying that for cystitis and anticholinergics, metabolic health seems to have a greater effect than obesity itself. In particular, in cystitis and anticholinergics, MHO showed lower HR than the control MHNO, which can be seen as strong evidence that obesity and metabolic health status should be separated and evaluated individually.

Several limitations of our study should be noted. First, there might be errors in the classification of metabolic health status according to disease code. The disease code cannot completely represent the patient's current disease status, and there may be an error as to whether each drug prescription accordingly occurs for each disease code. Second, because, our customized NIHS database had built randomly sampled 50% of the examinees who have undergone medical examinations, there may be errors in representing general populations during the study period. Also, the possibility of bias of healthy users should be considered due to the characteristics of the examinees who have undergone general medical examination.

In conclusion, obesity and metabolic health were individually or collaboratively involved in urological disorders related to voiding dysfunction. Metabolic healthy obesity needs to be distinguished in the diagnosis and treatment of urological disorders.

## Methods

### Study population and data source

**Figure 2:**
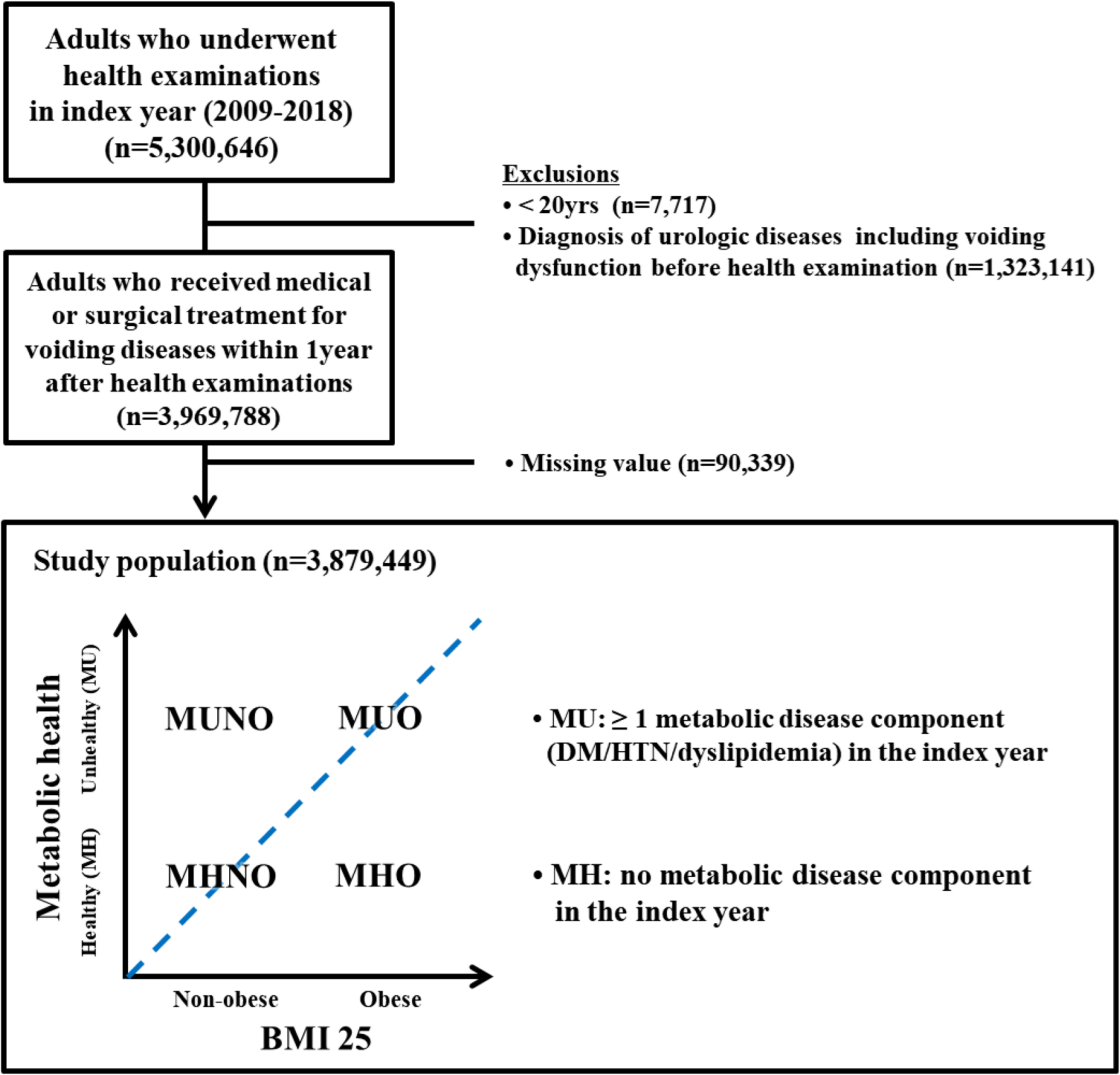
Flow diagram of study subjects. Of the 5,300,646 persons who received health examinations between 2009 and 2016, those aged < 20 years (n = 7,717) or those who were diagnosed with urologic diseases involving voiding dysfunction before the health examination (n = 1,323,141) were excluded. Within 1 year after the health examination, 3,969,788 patients were diagnosed with diseases related to voiding dysfunction and were treated medically or surgically. The final study population consisted of 3,879,449 subjects, except for 90,339 patients with missing values.

The National Health Insurance System (NHIS) maintains a comprehensive set of databases with health information on 50 million Koreans, including an eligibility database (with demographic factors including socioeconomic variables [including income], sex, age, and eligibility type), a health examination database (with data from general health examinations and lifestyle/behavior-related questionnaires), and a medical treatment database (containing information on claims made by medical service providers for medical expenses)^29–31^. The Health Insurance Review and Assessment (HIRA) service provides this information from their database, which includes information on insurance claims for approximately 97% of the Korean population. In the present study, we used a customized NHIS database including approximately 10% of the Korean population. The representativeness of the database was ensured by stratified random sampling. The index year was defined as the year of subjects’ first participation in the health examination. Of the 5,300,646 persons who received health examinations between 2009 and 2016, those who were < 20 years of age (n = 7,717) or had previously been diagnosed with urologic diseases involving voiding dysfunction (n = 1,323,141) were excluded. Within 1 year after the health examination, 3,969,788 patients were diagnosed with urological diseases related to voiding dysfunction and were treated medically or surgically. The final study population consisted of 3,879,449 subjects, with the further exclusion of 90,339 patients with missing values (Fig. 2). This study was developed in accordance with good clinical practice guidelines and the Declaration of Helsinki and was approved by the Hallym University Kangnam Sacred Heart Hospital Institution Review Board (HKS 2017-04-004) and exempted it from the requirement to obtain informed consent due to the use of anonymized and de-identified data.

### Measurements

As appropriate for Asian populations, obesity was defined using the BMI cutoff of 25 kg/m^2^. Samples were monitored according to the questionnaire before health examination of Koreans, family histories of cardiovascular disease, diabetes, and cancer were obtained using the questionnaire. Subjects were recorded as smoking status, and as drinking alcohol status based on the information obtained using the questionnaire. Regular exercise was defined as vigorous physical activity performed for at least 20 min, and subjects were classified based on the number of exercises per week.

### Metabolic health status and urological disorders related to voiding dysfunction

We classified to subjects by referring to previous study that defined metabolic health status^10^. Metabolic health status was defined on the basis of three disease components: diabetes, hypertension, and dyslipidemia. Diabetes was defined as (1) at least one claim annually for the prescription of an antidiabetic medication under International Classification of Disease, 10th Revision (ICD–10) codes E10–14, or (2) a fasting glucose measurement of ≥ 7 mmol/L. Hypertension was defined as the presence of one or more claim per year for the prescription of an antihypertensive agent (ICD-10 codes I10–I15) or a systolic/diastolic BP reading ≥ 140/90 mmHg. Dyslipidemia was defined based on the presence of one or more claim per year for the prescription of an antihyperlipidemic agent (ICD-10 code E78) or a total cholesterol measurement of ≥ 6.21 mmol/L. Participants with a BMI < 25 kg/m^2^ who developed at least one component of metabolic disease in the index year were defined as having the MUNO phenotype, and those with no components of metabolic disease were defined as having the MHNO phenotype. Analogously, participants with a BMI ≥ 25 kg/m^2^ were defined as having the MUO and MHO phenotypes, respectively, depending on the presence or absence of incident components of metabolic disease in the index yea. We analyzed data on the diagnostic codes according to ICD-10 classification such as benign prostatic hyperplasia (N400, N401, N402, N403, N408), chronic prostatitis (N411, N418), cystitis (N300, N301, N302, N303, N308, N309), neurogenic bladder (N31, N310, N311, N312, N318, N319), urinary incontinence (N393, N394), and cystocele (N811), and data for prescriptions of alpha-adrenergic antagonists, 5-alpha-reductase inhibitors, anticholinergic agents, and antidiuretic agents entered at all medical institutions during the observational period. We also analyzed data on the number of surgical procedures related to voiding dysfunction performed at all medical institutions during the observational period. Prostate operations included prostatectomy, transurethral resection, photoselective vaporization, and holmium laser enucleation. The anti-incontinence surgical procedures of interest included slings, suspensions, injectables, prosthetics, and reconstructions.

### Statistical analyses

Data are expressed as mean values (standard deviation), geometric mean values (95% confidence interval [CIs]), or percentages. Comparisons were made among the four groups according to BMI and metabolic health status using one-way analysis of variance or the chi-square tests. The hazard ratios (HRs) and 95% CIs were analyzed using Cox proportional hazards models, with the MHNO group as a reference. The proportional-hazard assumption was assessed using the logarithm of the cumulative hazards function based on Kaplan–Meier estimates for each group. Multivariable-adjusted proportional hazards models were constructed. A *P* value < 0.05 was considered to indicate statistical significance. Statistical analyses were performed using SAS version 9.3 (SAS Institute Inc., Cary, NC, USA).
